# Next-Generation Sequencing Reveals the Progression of COVID-19

**DOI:** 10.3389/fcimb.2021.632490

**Published:** 2021-03-11

**Authors:** Xiaomin Chen, Yutong Kang, Jing Luo, Kun Pang, Xin Xu, Jinyu Wu, Xiaokun Li, Shengwei Jin

**Affiliations:** ^1^ Institute of Genomic Medicine, Wenzhou Medical University, Wenzhou, China; ^2^ Wenzhou Key Laboratory of Sanitary Microbiology, Ministry of Education, Wenzhou, China; ^3^ Key Laboratory of Laboratory Medicine, Ministry of Education, Wenzhou, China; ^4^ School of Laboratory Medicine and Life Sciences, Wenzhou Medical University, Wenzhou, China; ^5^ Rheumatology Department, The First Affiliated Hospital of Wenzhou Medical University, Wenzhou, China; ^6^ Chemical Biology Research Center, School of Pharmaceutical Sciences, Wenzhou Medical University, Wenzhou, China; ^7^ Department of Anesthesia and Critical Care, Second Affiliated Hospital of Wenzhou Medical University, Wenzhou, China

**Keywords:** next-generation sequencing, SARS coronavirus 2 (SARS-CoV-2), origin, gut microbiota, angiotensin-converting enzyme 2 (ACE2)

## Abstract

The novel coronavirus SARS-CoV-2 (causing the disease COVID-19) has caused a highly transmissible and ongoing pandemic worldwide. Due to its rapid development, next-generation sequencing plays vital roles in many aspects. Here, we summarize the current knowledge on the origin and human transmission of SARS-CoV-2 based on NGS analysis. The ACE2 expression levels in various human tissues and relevant cells were compared to provide insights into the mechanism of SAS-CoV-2 infection. Gut microbiota dysbiosis observed by metagenome sequencing and the immunogenetics of COVID-19 patients according to single-cell sequencing analysis were also highlighted. Overall, the application of these sequencing techniques could be meaningful for finding novel intermediate SARS-CoV-2 hosts to block interspecies transmission. This information will further benefit SARS-CoV-2 diagnostic development and new therapeutic target discovery. The extensive application of NGS will provide powerful support for our fight against future public health emergencies.

## Introduction

In the end of 2019, the Chinese population was infected with a novel coronavirus, SARS coronavirus 2 (SARS-CoV-2, causing the disease COVID-19), which was identified from sequence-based analysis of isolates from the patients (Cui et al., 2019a). SARS-CoV-2 is the seventh known coronavirus that infects humans (Dallavilla et al., 2020). Phylogenetic analysis showed that SARS-CoV-2 belongs to the Sarbecovirus subtype of Betacoronavirus (Zhou et al., 2020b). SARS-CoV-2 uses angiotension-converting enzyme 2 (ACE2) as a receptor and primarily infects ciliated bronchial epithelial cells and type II pneumocytes (Kuhn et al., 2004). It has infected 78,939,299 individuals, with a mortality rate of 2.2%, across 109 countries as of the date 2020/12/24 (**Supplementary Figure 1**). The outbreaks of novel pneumonia add to evidence of the COVID-19 epidemic steadily growing by human-to-human transmission (Fang et al., 2020a). COVID-19 has very important clinical manifestations, such as high rates of transmission and mild to moderate clinical features, especially with more serious abnormalities found in the elderly (Yuen et al., 2020). Infected patients present with respiratory symptoms, such as fever, dry cough, and even dyspnea, as well as sometimes with digestive and other systemic manifestations, and some progress with severe acute respiratory syndrome or even death (Liu et al., 2020b).

The COVID-19 epidemic was declared a public health emergency of international concern. Since the outbreak of COVID-19, there has been considerable discussion on the origin and human-to-human transmission of the causative virus. Interestingly, ACE2 receptors are also reported to be expressed in the kidney and gastrointestinal tract, tissues known to harbor SARS-CoV (Harmer et al., 2002; Leung et al., 2003). Reports also suggest that SARS-CoV-2 RNA can be detected in the stool of some patients with COVID-19 (Wu et al., 2020b). Thus, there is the distinct possibility of involvement of the lung-gut axis caused by the gut microbiota, which is supported by the fact that some COVID-19 patients have diarrhea. A total of 273,606 new coronavirus-related genome sequences has been uploaded to the EpiFlu™ database (belong to GISAID) worldwide as of 22^nd^ December, 2020. Notably, next-generation sequencing (NGS) has been applied to the study of COVID-19 and has greatly promoted SARS-CoV-2 origin tracing. It is critical to explore the potential origins and mechanism of SARS-CoV-2 to control the spread of COVID-19 and further improve the therapeutic regimen.

## Next-Generation Sequencing Promotes SARS-CoV-2 Origin Tracing

**Figure 1:**
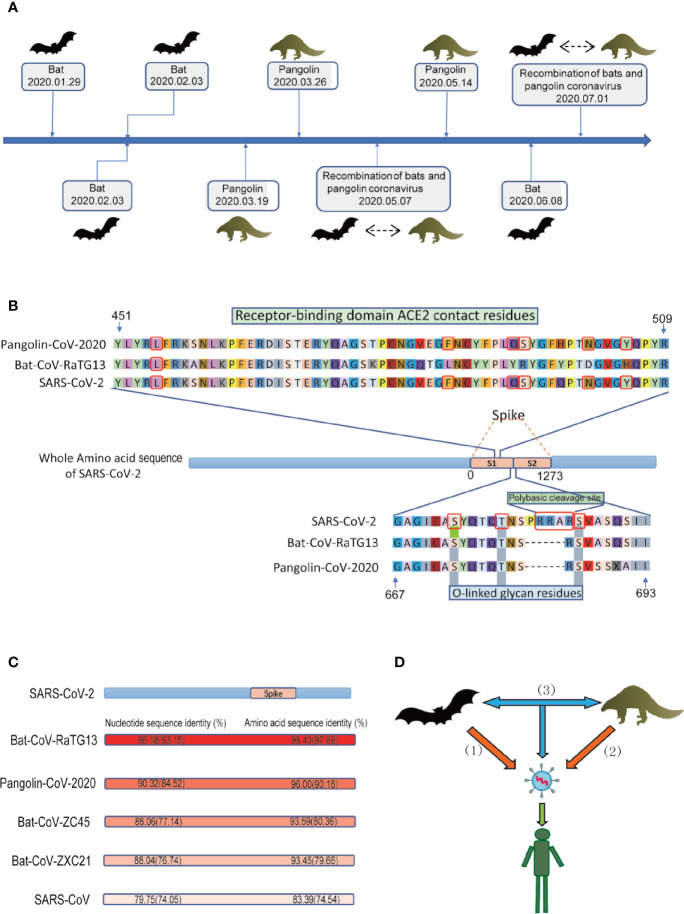
Conjectures on the origin of SARS-CoV-2. **(A)** The discovery of the potential animal origin of SARS-CoV-2 at different times, such as Bat-2020.01.29 (Lu et al., 2020a), Bat-2020.02.03 (Zhou et al., 2020e), Bat-2020.02.03 (Zhang et al., 2020b), Pangolin-2020.03.19 (Zhang et al., 2020c), Pangolin-2020.03.26 (Lam et al., 2020), Recombination of bats and pangolin coronavirus-2020.05.07 (Xiao et al., 2020), Pangolin-2020.05.14 (Liu et al., 2020b), Bat-2020.06.08 (Zhou et al., 2020b), Recombination of bats and pangolin coronavirus-2020.07.01 (Li et al., 2020c). **(B)** Features of the spike protein in human SARS-CoV-2 and related coronaviruses. Key residues in the spike protein that make contact with the ACE2 receptor are marked with blue boxes. Both the polybasic cleavage site and the three adjacent predicted O-linked glycans are unique to SARS-CoV-2. The six key RBD amino acids are indicated by red. **(C)** Nucleotide sequence identity and protein sequence identity among the whole genome including S gene (indicated by brackets) of some representative coronaviruses with SARS-CoV-2 **(D)** Three conjectures about the origin of SARS-CoV-2. i) SARS-CoV-2 originated in bats, ii) pangolins, and iii) *via* recombination events during the evolution of different animal coronaviruses.

NGS plays an important role in finding the origin and intermediate host of SARS-CoV-2. Hundreds of coronaviruses and SARS-CoV-2 genomes which were determined with NGS are publicly available for researchers to study the origin of SARS-CoV-2 (Woo et al., 2010; Zhou et al., 2020e). A small number of people speculate that SARS-CoV-2 may be man-made, but there is probably no basis for speculation about artificial SARS-CoV-2 (Dallavilla et al., 2020). Kristian G Andersen et al. clearly showed that SARS-CoV-2 is not a laboratory structure or a deliberately manipulated virus (Andersen et al., 2020). In addition, two notable genomic features of SARS-CoV-2 were identified by NGS (**Figure 1B**): Spike protein contains two subunits, S1 and S2, whose boundary position is at 685/686 of the amino acid sequence. S1 mainly contains receptor binding domain (RBD), which is responsible for the recognition of cell receptors. S2 contains the basic elements required for membrane fusion. The RBD in the spike protein is the most variable part of the coronavirus genome. Six RBD amino acids have been shown to be critical for binding to ACE2 receptors and for defining the host range of SARS-CoV-like viruses (Wan et al., 2020); additionally, the spike protein of SARS-CoV-2 inserts 12 nucleotides with a functional multibase (furin) cleavage site (RRAR) at the S1-S2 boundary, which also leads to the expected three O-linked glycans around the site (Andersen et al., 2020).

During 2020.01.29-2020.02.03, Lu et al. performed NGS of bronchoalveolar lavage fluid samples (n=9) (Lu et al., 2020a). Zhou et al. used NGS to obtain the viruses from seven patients with severe pneumonia and carried out full-length sequencing on an RNA sample of *Rhinolophus affinis* (Global Initiative on Sharing All Influenza Data, GISAID). The metagenomic analysis of the bat sample (*Rhinolophus affinis*) showed that the most closely related virus to SARS-CoV-2 is RaTG13, with an identity of 96.2% (**Figure 1**) (Zhou et al., 2020e). Similarly, Zhang et al’s phylogenomic analysis of the released genomic data of 27 isolates of SARS-CoV-2 showed that bat coronavirus and SARS-CoV-2 probably have a common ancestor (Hu et al., 2018). SARS-CoV-2 was first isolated from street vendors selling wild animals and mammals (Zhang et al., 2020b). On 2020.06.08, Zhou et al. reported a coronavirus named RmYN02 (identified from a metagenomic analysis of 227 bats) have 93.3% nucleotide identity with SARS-CoV-2 in the complete genome. However, RmYN02 has a low sequence identity with SARS-CoV-2 in the RBD (61.3%) (Zhou et al., 2020b). All these studies indicated that SARS-CoV-2 may have originated from bats.

During 2020.03–2020.05, Zhang et al. used reference genome of the *Manis javanica* (SRA), SARS-CoV-2, Bat-CoV-RaTG13 (NGDC), and 2,845 coronavirus from ViPR for genome analysis. In terms of sequence identity, in addition to Bat-CoV-RaTG13, Pangolin-CoV-2020 is the coronavirus most closely related to SARS-CoV-2 (**Figure 1C**). Because the six RBD amino acids residues (L, F, Q, S, N, and Y, **Figure 1B**) on the S1 protein of Pangolin-CoV-2020 are exactly the same as those of SARS-CoV-2 ((Wan et al., 2020), Receptor Recognition by the Novel Coronavirus from Wuhan: an Analysis Based on Decade-Long Structural Studies of SARS Coronavirus), while Bat-CoV-RaTG13 has only one amino acid residue that is the same as on the six RBD residues in SARS-CoV-2. Thus, they proposed that the pangolin is more likely than the bat to be the origin of SARS-CoV-2. However, Pangolin-CoV-2020 and Bat-CoV-RaTG13 have lost the key polybasic cleavage site (RRAR) at the junction of S1 and S2 (**Figure 1B**), the two subunits of the spike of SARS-CoV-2 (Zhang et al., 2020c). Furthermore, a similar debatable conclusion was also obtained (Liu et al., 2020b). In addition, NGS of frozen tissues (lungs, intestine, and blood) collected from 18 Malayan pangolins (*Manis javanica*) identified pangolin-associated coronaviruses as belonging to two SARS-CoV-2-associated coronavirus sublines, one of which has a strong similarity to SARS-CoV-2 in the RBD (Lam et al., 2020). These discoveries only suggest that pangolins may be the origin of SARS-CoV-2-like viruses.

During 2020.05–2020.07, Xiao et al. identified 34 highly related contigs in a set of pangolin viral metagenomes based on BLAST results. Subsequently, metagenomic sequencing of the lung tissue of four Chinese pangolins (*Manis pentadactyla*) and 25 Malayan pangolins (*Manis javanica*) showed surprising results. The sequence identity of the Pangolin-CoV genome, comparing with SARS-CoV-2 and Bat SARS-related (SARSr-CoV) RaTG13 is 80%–98% excluding the S gene, while the identity is 80%–91% including the S gene. Further comparison and analysis of the S gene revealed that these genomes may have undergone recombination events. For example, Pangolin-CoV is more similar to Bat SARSr-CoV ZXC21 and Bat SARSr-CoV ZC45 in the region of nucleotides 1-914, while in the remaining part, it is more similar to SARS-CoV-2-like and Bat-CoV-RaTG13. According to the existing genome comparison, SARS-CoV-2 may have originated from the recombination of Pangolin-CoV-like viruses and Bat-CoV-RaTG13-like viruses (Xiao et al., 2020). Liu et al. also obtained similar results showing that a recombination event could have occurred during the evolution of these coronaviruses (Liu et al., 2020c). Li et al. analyzed 43 complete coronavirus genome sequences (Li et al., 2020c), including coronaviruses from bats, pangolins and humans. Their study showed that the currently sampled pangolin coronaviruses are unlikely to be precursors of SARS-CoV-2, but these sequences contain the receptor-binding motif most likely to bind to human ACE2. Excluding S genes, SARS-CoV-2 has the greatest genetic similarity with RaTG13. In addition, researchers found recombination fracture sites before and after the ACE2 RBD in SARS-CoV-2. Therefore, they think that SARS-CoV-2 was produced by the selection and recombination of bat and pangolin coronaviruses.

Recently, the SARS-CoV-2 epidemic broke out in a newly developed market in Beijing. Relevant agencies have detected SARS-CoV-2 in the cutting board of imported salmon. Researchers discovered 3 new viruses in wild and farmed salmon in September 2019 by metatranscriptomic sequencing of dead and moribund cultured Chinook salmon (Mordecai et al., 2019). We analyzed the nucleotide sequences of SARS-CoV-2 (NCBI, GenBank: MN908947.3) and Pacific salmon nidovirus (PsNV, GenBank: MK611985.1), one from the three new viruses, and found only 22%–35% identity in partial protein sequence (**Supplementary Table 1**), and PsNV does not have key amino acid residues similar to the SARS-CoV-2 spike protein, which can bind to human ACE2. Therefore, the possibility that salmon is the origin or intermediate host of SARS-CoV-2 is low based on the genome data obtained by NGS. This result further supports the evidence that SARS-CoV-2 infects only mammals since it belongs to the Coronaviridae family, Betacoronavirus (Cui et al., 2019a).

Using NGS technology, the genome sequence information of unknown viruses can be obtained for the first time. This information provides the possibility to quickly trace the potential origin of novel SARS-CoV-2, which is vital to blocking interspecies transmission. However, this method is limited by the number of known sequences. At present, scientists and researchers upload whole genomic sequences of SARS-CoV-2 at GISAID EpiFlu™, and the number of genomes is still increasing. It may take time due to the massive diversity and evolution of the virus. We still believe that NGS technology will greatly promote the process of tracing the origin of SARS-CoV-2.

## Next-Generation Sequencing Promotes Tracing Interpersonal Transmission of SARS-CoV-2

SARS-CoV-2 is a positive-sense RNA virus belonging to the genus Betacoronavirus. Recent genotyping analysis of the SARS-CoV-2 population revealed a high frequency of mutations in various essential genes encoding S protein, N protein, and RNA polymerase (Yin, 2020). SARS-Cov-2 may first indirectly control mitochondria through ACE2 regulation of mitochondrial function. Once the virus enters the host cell, open reading frame (ORF) (e.g., ORF-9B) can directly manipulate mitochondrial function to evade the immune function of host cells and promote viral replication, causing COVID-19 disease (Singh et al., 2020). The evolutionary pattern of SARS-CoV-2 with highly frequent mutations can be observed in a short time using NGS. The monitoring of SARS-CoV-2’s evolutionary patterns and spread dynamics by genome sequencing is essential for controlling and preventing COVID-19. High-resolution genomic epidemiology has become an effective tool for public health surveillance and disease control, and the large number of the real-time genome sequencing efforts for SARS-CoV-2 were triggered by the current COVID-19 pandemic (Sintchenko et al., 2009; Gardy and Loman, 2018; Grubaugh et al., 2019b). Analysis of the genetic sequence data of the pandemic SARS-CoV-2 can provide insights into the origin of the epidemic, global transmission, and epidemiological history.

The outbreak of COVID-19 started in mid-December 2019 in Wuhan, China, and rapidly spread throughout China. The haplotype evolution network can be utilized for recovering the direction of human-to-human transmission in a local area and spread in a larger area. A study conducted metagenomic NGS on 10 samples from COVID-19 patients in Hubei. Ten newly sequenced SARS-CoV-2 genomes and 136 genomes from the GISAID database were classified as 58 haplotypes. The network indicated that virus samples corresponding to the original haplotype found in patients living near the Huanan Seafood Wholesale Market. Although the patients had a high probability of subconscious contact with the market, it is impossible to draw a conclusion based on this clue regarding whether the market is the origin center of SARS-CoV-2. Among them, 16 genomes from the Huanan Seafood Wholesale Market were assigned to 10 haplotypes, indicating that the market experienced a circulating infection in the short term, which caused the outbreak of SARS-CoV-2 in Wuhan and other regions (Fang et al., 2020a).

Using both metagenomic sequencing and tiling amplicon approaches, viral genome sequences were generated from 53 patients in Guangdong. Combined genomic data and epidemiological data showed that the SARS-CoV-2 sequences in Guangdong Province are interspersed with virus clades from other provinces in China and other countries, indicating that most of the detected cases are related to travel, not local communities. Following the discovery of the first COVID-19 case in early January, most infections were attributed to viruses imported from elsewhere. The size and duration of the local transmission chain is limited (Lu et al., 2020a).

Since the emergence of COVID-19 in Asia at the end of last year, the disease has spread to all continents except Antarctica (Rodriguez-Morales et al., 2020). To reveal the source of SARS-CoV-2 introduction and the patterns of transmission in the United States, Fauver et al. sequenced the nine viral genomes of patients with COVID-19 reported early in Connecticut using the amplicon sequencing approach ‘‘PrimalSeq” (Grubaugh et al., 2019a), using ‘‘genomic epidemiology’’ to identify the likely sources of SARS-CoV-2 in Connecticut. The combined results of genomic epidemiology and travel pattern analysis indicated that family transmission has become an important source of new SARS-CoV-2 infection. The outbreak on the east coast (Connecticut) is related to the outbreak on the west coast (Washington), which indicates that transcontinental transmission has already occurred.

To investigate the genomic epidemiology and genetic diversity of SARS-CoV-2 in Northern California, 29 samples from patients diagnosed with COVID-19 infection were collected for whole-genome sequencing. Phylogenetic analysis showed that there are at least eight different SARS-CoV-2 lineages, indicating that the virus has been independently introduced into the state many times. To date, the cryptic transmission of SARS-CoV-2 in Northern California has been characterized by multiple transmission chains, whose origin is a unique introduction from international and interstate travel, rather than the widespread community transmission of a single main lineage (Deng et al., 2020).

To prospectively monitor the spread of incidents in New South Wales, Australia, SARS-CoV-2 was extracted from 209 patients diagnosed with COVID-19 infection for whole-genome sequencing from January 2020 to March 2020. Computational agent-based modeling and high-resolution SARS-CoV-2 genome surveillance were used to understand the evolution of this novel virus strain and the local transmission chain in the community. The 209 New South Wales SARS-CoV-2 genomes are scattered throughout the global SARS-CoV-2 phylogeny. Most genomes from imported New South Wales cases grouped with viral sequences from the origin country. The major genomic lineage is dominated by genomes from Australia, which are considered to be imported from Iran (Rockett et al., 2020).

A study collected genome data of 93 SARS-CoV-2 isolates covering 12 countries on 4 continents from the GISAID EpiFlu^TM^ database (access date 12 February 2020). All the genomes were classified as 58 haplotypes (H1~H58). The results showed that H13 and H38 are the ancestral haplotypes of SARS-CoV-2. The virus samples corresponding to these two haplotypes were from patients from Shenzhen (the first case in Guangdong) and patients from Washington state (the first case in the United States). The potentially specific directions of human-to-human transmission were recovered by this approach, such as in the England family (H28 → H29), Shenzhen family (H13 → H14), Queensland tour group (H25 → H26), and Japanese group (H53 → H52). Phylogenetic network analysis showed that the source of SARS-CoV-2 in the Huanan Seafood Wholesale Market was imported from elsewhere (Yu et al., 2020).

**Figure 2:**
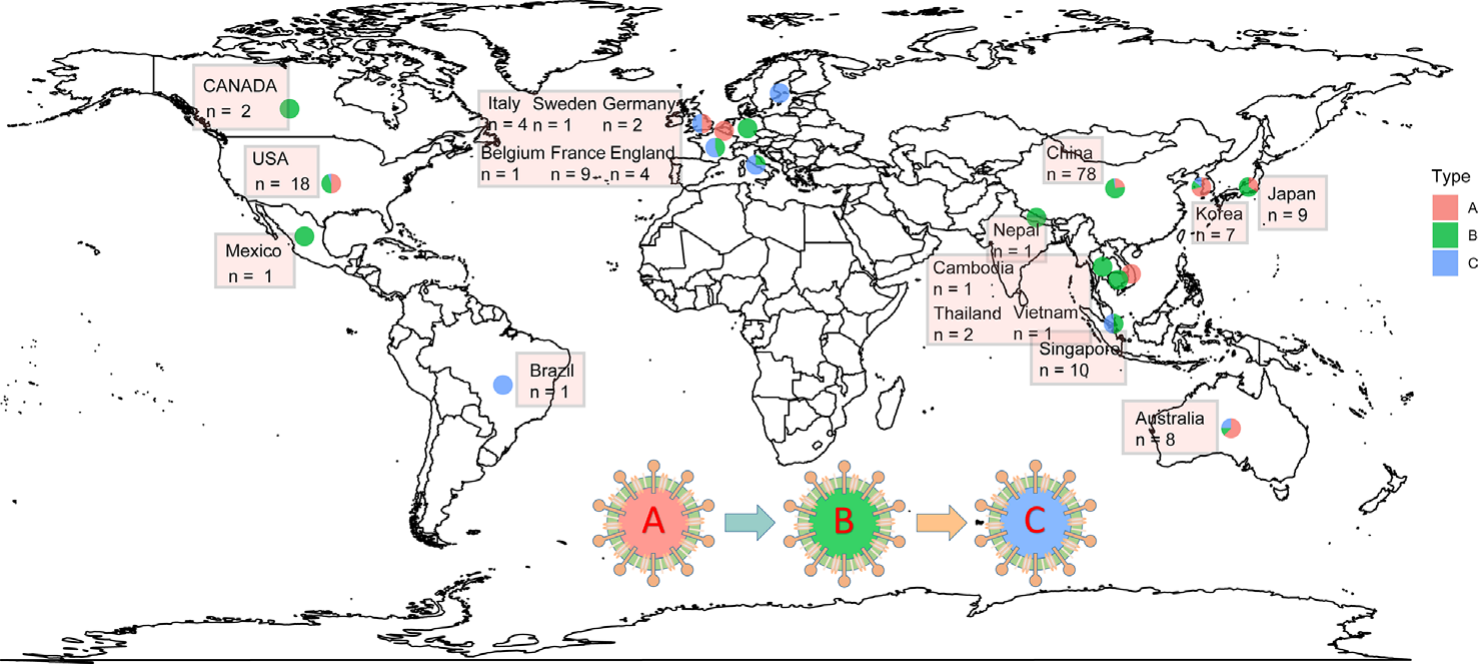
The distribution of SARS-CoV-2 A\B\C types in some countries worldwide. The data were collected from 2019.12.24 to 2020.03.04. The type A was indicated by red; type B was indicated by green; and type C was indicated by green.

Phylogenetics is very important to solve various biological problems, such as the relationship between species or genes, the origin and spread of viral infections, and the demographic changes and migration patterns of species (Avise and Wollenberg, 1997; Grenfell et al., 2004; Li and Durbin, 2011). The NGS technology and the rapid accumulation of genome sequence data have made phylogeny an indispensable tool for all branches of biology (Yang and Rannala, 2012). Phylogenetic network analysis of 160 complete SARS-CoV-2 genome sequences collected from around the world from December 24, 2019 to March 4, 2020 revealed that there are three distinct but closely related variants of SARS-CoV-2 called A, B, and C (**Figure 2**). Based on the bat outgroup coronavirus, A is an ancestral type. Data analysis found that the first type A virus was from an American individual living in Wuhan. At the same time, the type A virus itself was also detected in a large number of infected people in the United States and Australia, but surprisingly, type A is not the main type of virus in Wuhan city. The main virus type of patients in Wuhan is type B, and it has infected patients throughout East Asia. Type B virus is immunologically or environmentally suitable for most populations in East Asia, and if there is no further mutation, it will not spread much outside the region. People in Wuhan also exhibited type A infection, and the earliest cases were in American individuals. Type C virus is the main European type and was found in early patients from France, Italy, Sweden, and the United Kingdom. Type C virus did not appear in the Chinese patient samples in the study, but it was found in Singapore, Hong Kong, and South Korea (Forster et al., 2020).

The use of NGS technology has become a widely accepted method for outbreak tracking and genomic epidemiology in molecular typing in microbiology laboratories. Detecting new mutations in SARS-CoV-2 allows researchers to reconstruct unknown routes of infection and provide a molecular basis for SARS-CoV-2 vaccine design, drug discovery, and diagnostic development, which is a key technology in genomic epidemiology (Lourenco et al., 2020). Monitoring the SARS-CoV-2 genome helps to achieve a more effective COVID-19 control strategy and to investigate cases with unclear sources of infection within a short turnaround time (Sintchenko and Holmes, 2015; Deng et al., 2020; Lu et al., 2020a; Lu et al., 2020b). The international sharing of SARS-CoV-2 genomic data collected by researchers and public health microbiology service providers enhances the power of such surveillance (Dos et al., 2018; Hadfield et al., 2018).

## SARS-CoV-2-Susceptible Organs With High Expression of ACE2 Determined by Next-Generation Sequencing

Over the past years, RNA sequencing technology has achieved dynamic improvement of genomes, including DNA and RNA quantitative and qualitative changes, which were widely applied into various genomic measurements especially transcriptomics studies (Consortium et al., 2007). However, ordinary RNA-seq was also confronted with several challenges including limited sequencing depth for low-abundance transcripts and ineffaceable influence of interferential cells’ gene expression in tissues (Levin et al., 2009). Similar to the process of RNA-seq, scRNA-seq also have to undergo some required steps including RNA extraction, transcription into first-strand cDNA, second-strand synthesis, and cDNA amplification (Islam et al., 2014). Moreover, through preliminary single-cell extraction, scRNA-seq could overcome the influence of heterogeneous cells expression in tissues and accomplish various analysis including cell type identification, cell trajectory inference, cell hierarchy reconstruction, and other potential applications (Hwang et al., 2018). Hence, NGS technology could be applied to identify relative gene expression and detect special cell type in various diseases.

**Figure 3:**
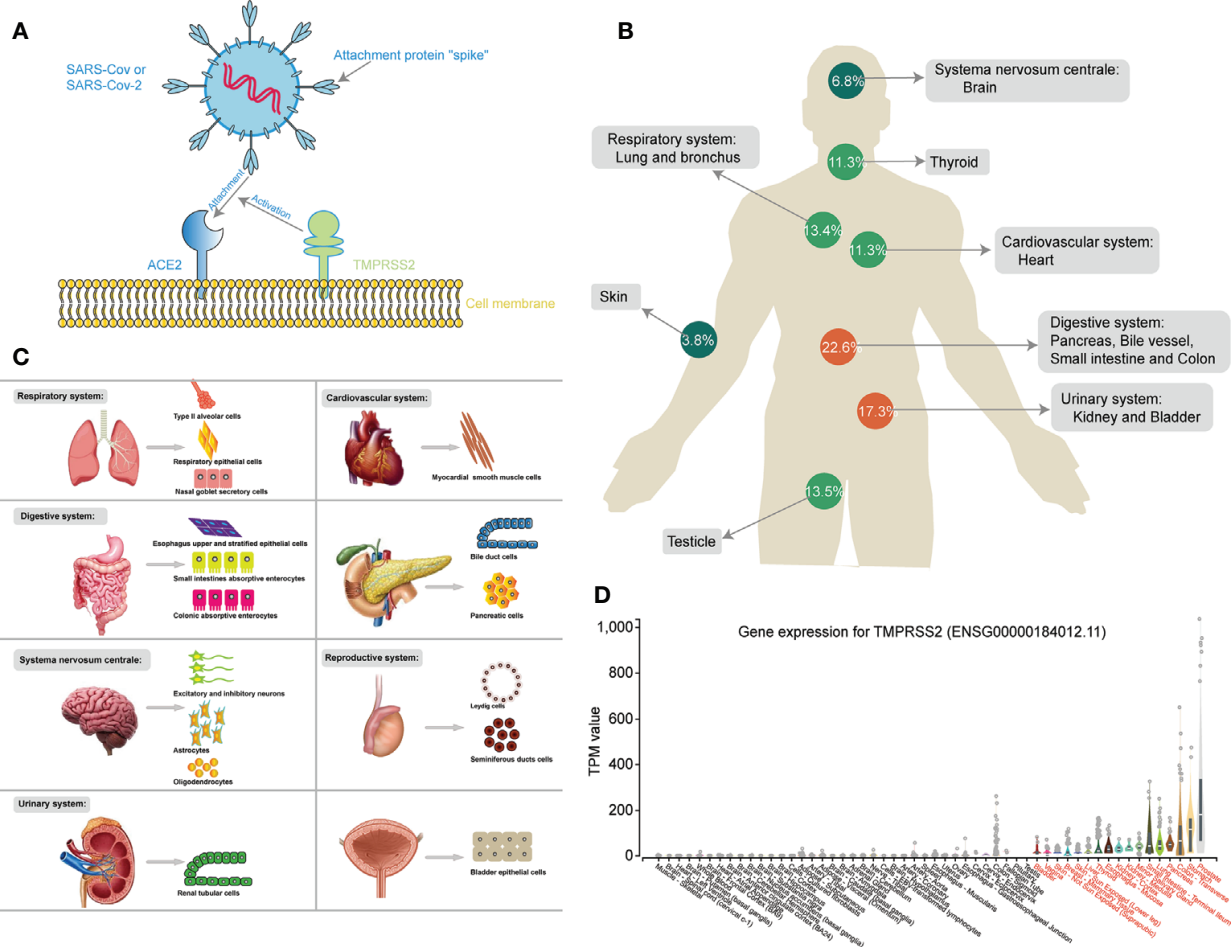
**(A)** Schematic chart showing the mechanism of SARS-CoV-2 binding to the ACE2 receptor, assisted by TMPRSS2. **(B)** The human figure displays different expression levels of ACE2 in different organs. **(C)** The cell maps show different susceptible cells of different susceptible organs during SARS-CoV-2 infection. **(D)** The box diagram showing the different expression of TMPRSS2 in different human tissues.

NGS technology was applied at either the molecular or protein level to further investigate the mechanism of SAS-CoV-2 infection. Only when bound to cell surface receptors can the virus enter the target cells for further replication, which is the prerequisite of coronavirus infection (Li, 2016). ACE2 is a cell surface protein that is expressed in the heart, blood vessels, kidney, and especially lung type II alveolar (AT2) epithelial cells (Richardson et al., 2020a). Two teams independently reported the discovery that ACE2 could serve as the receptor for SAS-CoV-2 (Xu et al., 2020b; Zhou et al., 2020d). Furthermore, SARS-CoV-2 could enhance infection by exploiting species-specific interferon-driven upregulation of ACE2 (Ziegler et al., 2020). Therefore, the different organs, tissues and cells with high ACE2 expression are considered targets with potential high infection risk for SARS-CoV-2. In **Figure 3A**, the mechanism for the binding of ACE2 and SARS-CoV-2 through spike protein, assisted by TMPRSS2, was clearly studied by massive researches (Hoffmann et al., 2020).

Specific comorbidities were found to increase the risk of infection with worse lung injury and death in COVID-19 and hypertension, obesity, and diabetes were identified as the most common comorbidities in COVID-19 according to a clinical research of 5,700 patients in the New York City Area (Richardson et al., 2020b). Moreover, the expression imbalance of ACE2 was found significantly associated with multiple comorbidities in COVID-19. A Mendelian randomization analysis revealed the high-expression level of ACE2 was causally associated with diabetes, which might interpret the high risk to COVID-19 in diabetic patients (Rao et al., 2020). In addition, Sammy et al. identified higher expression of ACE2 in visceral and subcutaneous adipose tissue than that in lung tissues, providing with a possible interpretation for susceptibility of infection in obese patients (Al-Benna, 2020). In China, about 23% of hypertensive COVID-19 patients were reported with 6% case fatality rate (Ma et al., 2020) and hypotensors, especially ACE2 inhibitors, and angiotensin receptor blockers (ARBs), were frequently used to control the levels of blood pressure, which also upregulated the expression of ACE2 receptor and leaded to increasing susceptibility to SARS-CoV-2 infection (Fang et al., 2020b). Further, other common comorbidities including chronic obstructive pulmonary disease (COPD), asthma, and cardiovascular disease (CVD) were also found more severe symptom and prognosis in COVID-19 with high expression of ACE2 (Contoli et al., 2006; Shi et al., 2020; Wan et al., 2020). Here, we summarize the expression features of ACE2 in the respiratory, cardiovascular, digestive, urinary, and reproductive systems through RNA sequencing (RNA-seq) and single-cell RNA sequencing (scRNA-seq) to investigate susceptibility to COVID-19.

Respiratory symptoms are usually the initial clinical presentation of COVID-19, including fever, chest pain, shortness of breath, dry cough, and fatigue, suggesting that the respiratory system is the most common target organ of SARS-CoV-2 infection (Ge et al., 2020; Zhou et al., 2020c). A previous study investigated the mRNA and protein expression of ACE2 from different human tissues by RT-qPCR and immunohistochemistry and found that ACE2 was concentrated in lung alveolar epithelial cells and enterocytes of the small intestine (Harmer et al., 2002; Hamming et al., 2004). Moreover, Xin Zou et al. analyzed scRNA-seq datasets of the human respiratory system, including the nasal mucosa, respiratory track, and bronchus, using lung tissues and airway epithelial cells. Lung ACE2 was further identified to be highly expressed in AT2 and respiratory epithelial cells, while nasal and bronchial cells showed low ACE2 expression (Zou et al., 2020). Zhao’s study also found that ACE2 expression in AT2 cells significantly upregulated other genes relevant to viral reproduction and transmission through scRNA-seq (Zhao et al., 2020). Furthermore, lung type II pneumocytes and nasal goblet secretory cells were identified as ACE2 and TMPRSS2-coexpressing cells in both humans and nonhuman primates *via* scRNA-seq analysis. Based on the above studies, AT2, respiratory epithelial, and nasal goblet secretory cells with high expression of ACE2 based on NGS technology probably have a high SARS-CoV-2 infection risk.

Xin Zou et al. investigated scRNA-seq datasets from the esophagus, stomach, ileum, and liver and found extremely high ACE2 expression in epithelial cells of the ileal esophagus, which is consistent with the results of histopathology and immunohistochemical staining by (Hamming et al., 2004; Zou et al., 2020). Moreover, the distribution of ACE in the digestive system was systematically analyzed *via* scRNA-seq from surgically resected digestive organs tissues in Xu’s study (Zhang et al., 2020a). High-level expression of ACE2 was identified not only in upper and stratified epithelial cells of the esophagus but also in absorptive enterocytes from the ileum and colon, consistent with previous studies (Hamming et al., 2004). Regarding other organs of the digestive system, it was reported by Liu et al. that the mRNA level of ACE2 was higher in the pancreas than in the lung and that ACE2 was expressed in both the exocrine glands and islets *via* scRNA-seq (Liu et al., 2020a). In addition, scRNA-seq analysis was also applied to identify specific ACE2 expression in cholangiocytes rather than hepatocytes, suggesting that liver abnormalities might be caused by cholangiocyte dysfunction (Chai et al., 2020). All these studies identified the specific cellular susceptibility to SARS-CoV-2 infection in the digestive system according to NGS and scRNA-seq technology, especially in esophageal upper and stratified epithelial cells, absorptive enterocytes from the ileum and colon, exocrine glands, islets, and cholangiocytes.

It has been reported that patients with SARS-CoV-2 infection have a high proportion of acute and chronic cardiac injury, based on clinical studies (Wu et al., 2020a; Zheng et al., 2020). Moreover, Li et al. analyzed the mRNA expression of ACE2 in 13 different human organs from the GTEx database with NGS and found that ACE2 expression levels were high in the small intestine, testes, kidneys, heart, thyroid, and adipose tissues but relatively low in the blood, spleen, bone marrow, brain, blood vessels, and muscle (Li et al., 2020b). Regarding specific cells, ACE2 was identified to be overexpressed in massive myocardial cells through scRNA-seq using cardiac cells from human embryos (Zou et al., 2020). Additionally, scRNA-seq also demonstrated high expression of ACE2 in specific cells of human fetal hearts, including cardiomyocytes (CM), macrophages, smooth muscle cells, and pericytes (SMC/Peri) (Cui et al., 2019b).

Using scRNA-seq from tissues of kidney transplant and bladder tissues homogenate, high expression of ACE2 was confirmed in kidney proximal tubule (PT) cells and bladder urothelial cells in Xin Zou’s study, indicating that the kidney and bladder might also be the target organs for SARS-CoV-2 infection (Zou et al., 2020). Moreover, quite similar results showing that overexpressed ACE2 was identified in renal tubular cells of patients with COVID-19 were also validated in another study (Li et al., 2020d). In addition, both Fan’s and Wang’s studies (Fan et al., 2020; Wang and Xu, 2020) also found that ACE2 was highly expressed in Leydig cells and cells in seminiferous ducts in the testis, indicating that the urinary and reproductive systems are susceptible to SARS-CoV-2 infection due to high levels of ACE2.

In addition to atypical pneumonia, the central nervous system (CNS) symptoms of COVID-19 patients have been observed in the clinic, including dizziness, headache, impaired consciousness, acute cerebrovascular disease, ataxia, and epilepsy (Li et al., 2020d; Mao et al., 2020). Massive microarray, RNA-seq and scRNA-seq analyses have indicated that nuclear expression of ACE2 was identified in many neurons and some non-neuron cells in the human middle temporal gyrus and posterior cingulate cortex. These expression features of ACE2 were also validated in a mouse model using brain tissues (Chen et al., 2020).

Aside the CNS, several specific organs, such as the skin, thyroid, and adrenal gland, also exhibited high expression of ACE2, as detected by scRNA-seq analysis (Li et al., 2020b). In particular, Li et al. demonstrated that ACE2 is highly expressed in maternal-fetal interface cells, including stromal cells and perivascular cells of the decidua and cytotrophoblasts and syncytiotrophoblasts in the placenta. Moreover, high expression of ACE2 was also validated in human fetal organs, including the heart, liver, and lung but not the kidney (Li et al., 2020a).

Furthermore, Bian et al. directly detected SARS-CoV-2 RNA in the stomach, breast, testes, spleen, heart, hilar lymph nodes, liver, gallbladder, kidney, nasopharyngeal mucosa, oral mucosa and skin through virus nucleic acid RT-qPCR detection, immunohistochemical staining, and electron microscopy from 37 systematic autopsy and 54 percutaneous multiple organ biopsy cases, consistent with the results of RNA-seq (Wu and Team, 2020). On a cautionary note, the NGS data from Genotype-Tissue Expression (GTEx) project also clear exhibited the mRNA expression of TMPRSS2 in different human tissues (**Figure 3D**) (Consortium, 2013). Similar to the expression of ACE2, TMPRSS2 was not only high-expressed in respiratory (lungs) and digestive system (stomach, transverse-colon, pancreas, small intestine, esophagus -mucosa, and liver), but also up-regulated in urinary system (kidney-medulla and cortex, and bladder) and other glandular tissues (prostate, thyroid and mammary tissue), perhaps contributing to one explanation for non-respiratory symptoms in COVID-19 patients.

In conclusion, NGS and scRNA-seq were applied to reveal the expression features of ACE2 in different organ systems and specific cells after SARS-CoV-2 infection. The ratio of ACE2 expression in different organ systems was clearly exhibited, and the respiratory, digestive, cardiovascular, urinary, and reproductive systems were identified as high-risk organs with relatively high expression of ACE2, while relatively low expression of ACE2 was found in the CNS and skin (**Figure 3B**). Moreover, a susceptibility cell map of SARS-CoV-2 infection with high ACE2 expression is displayed in a systemic multiorgan depiction (**Figure 3C**). These findings will further benefit SARS-CoV-2 diagnostics and new therapeutic target identification.

## The Changes in the Intestinal Flora Revealed by Metagenomics Sequencing

In addition to common clinical symptoms in the lungs, some COVID-19 patients have also developed gastrointestinal symptoms, such as diarrhea, nausea, and vomiting (Chen et al., 2020; Gu et al., 2020a; Wang et al., 2020a). It was recently reported that COVID-19 RNA was found in the fecal samples and anal swabs of infected patients (Wu et al., 2020b; Zhou et al., 2020a). Interestingly, intestinal epithelial cells, particularly the crypt surface of small intestine epithelial cells, also express ACE2 receptors. Researchers also found that ACE2 mutations may reduce the expression of antimicrobial peptides and change the composition of intestinal microbes (Hashimoto et al., 2012). In addition, the intestinal flora has been shown to affect lung health through important cross-talk between the intestinal flora and lungs, which is known as the gut-lung axis (Keely et al., 2012). The gut-lung axis is considered to be bidirectional, which means that endotoxins and microbial metabolites can affect the lungs through blood circulation, and inflammation of the lungs may change the gut microecology (Dumas et al., 2018). It is also known that respiratory viral infections can disturb the intestinal flora, such as influenza and respiratory syncytial virus (RSV) (Groves et al., 2018). This fact raises the interesting possibility that COVID-19 may also affect the intestinal flora.

The intestinal microbiota is a dynamic ecological community and can regulate promote or suppress response by invading viruses (Li et al., 2019). Prior studies have suggested that respiratory viral infections are associated with alterations of the gut microbiota, which made patients more susceptible to secondary bacterial infections (Hanada et al., 2018; Yildiz et al., 2018). A preliminary study using shotgun metagenomic sequencing of 15 patients with COVID-19 found that the fecal microbiome of COVID-19 patients had continuous changes during hospitalization compared with that of the control group. Many pathogens and opportunistic pathogens were enriched in the intestinal microbiome of patients with COVID-19, including Clostridium, *Bacteroides nordii*, and *Actinomyces viscosus*. Research on the oral and upper respiratory microbiome of patients with the COVID-19 also found an increase in the abundance of the opportunistic pathogen *Actinomyces viscosus* (Habib et al., 2018). Even after the clearance of SARS-CoV-2 (as determined by a throat swab) and relief of respiratory symptoms, depleted symbionts, and intestinal dysbiosis persisted. Correlation analysis between fecal microbe baseline abundance and COVID-19 severity showed that *Coprobacillus*, *Clostridium ramosum*, and *Clostridium hathewayi* were significantly positively correlated with COVID-19 severity, whereas *Faecalibacterium prausnitzii* (an anti-inflammatory bacteria) was inversely related to the severity (mild, moderate, severe, or critical) of disease (Zuo et al., 2020).

A recent study compared the fecal microbiome of COVID-19 patients with that of healthy controls (HCs) and H1N1 patients and obtained results similar to those of Tao et al. (Gu et al., 2020b) (**Table 1**). Compared with that in the gut microbiome of HCs, the relative abundance of opportunistic pathogens (such as Streptococcus, Rothia, Veillonella, and Actinomycetes) in the gut microbiome of COVID-19 patients was significantly higher, and the relative abundance of beneficial symbiotic bacteria was lower. Compared with COVID-19 patients, H1N1 patients showed lower diversity and different overall microbial compositions.

**Table 1:** The change of gut microbiome in COVID-19 patients (Gu et al., 2020b).

Title	Subject	Sample	Sequencing method	Conclusion
Alterations in Gut Microbiota of Patients With COVID-19 During Time of Hospitalization	15 COVID-19 patients	fecal samples	shotgun metagenomic sequencing	enrichment of opportunistic pathogens and depletion of beneficial symbionts
	Six community-acquired pneumonia patients			
	15 healthy controls			
Alterations of the Gut Microbiota in Patients with COVID-19 or H1N1 Influenza	30 COVID-19 patients	fecal samples	16sRNA	
	24 influenza A patients			
	30 healthy controls			

Intestinal flora regulation is very important for improving the immune system, preventing viral infections and reducing clinical signs. Some patients with COVID-19 show dysbiosis of the intestinal flora, and the abundance of probiotics, such as Lactobacillus and Bifidobacterium, is reduced. The use of prebiotics or probiotics may help to regulate intestinal flora balance and reduce the risk of secondary infections caused by bacterial translocation after coronavirus infection (Xu et al., 2020a). In addition, a fiber-rich diet not only changes the intestinal flora but also affects lung immunity (Trompette et al., 2014). Circulating short-chain fatty acids (SCFAs) produced during microbial metabolism of fermentable fibers in the diet may have a mitigating effect on lung allergic inflammation (Trompette et al., 2014). Intestinal flora diversity decreases in old age, and COVID-19 is fatal mainly in elderly patients, which again shows that the intestinal flora may play a role in the disease (Nagpal et al., 2018).

## Immunogenetics of COVID-19 Revealed Through Single-Cell RNA Sequencing

Acute respiratory distress syndrome (ARDS) and multiple organ failure have been reported as the major causes of death due to SARS-CoV-2 infection, associated with high levels of circulating cytokines and substantial mononuclear cell infiltration in the heart, lungs, kidneys, and spleen through autopsy analysis (Diao et al., 2020b; Mehta et al., 2020; Xu et al., 2020c). The mechanism of the immune response is complex during SARS-CoV-2 infection, and Ronny et al. identified two distinct immunopathological profiles. One is high expression of interferon-stimulated genes (ISGs) and cytokines and high viral loads with limited pulmonary damage, and the other is severely damaged lungs with downregulated ISGs, low viral loads, and abundant immune infiltrates (Nienhold et al., 2020). A better understanding of the immune cells associated with differential responses in SARS-CoV-2-infected patients will be immensely helpful to better identifying therapeutic targets. However, the concrete mechanisms of the key immune cell subset changes remain unclear. Here, we summarized the major distinct immune cell compositions and states of different degrees of SARS-CoV-2 infection by scRNA-seq technology. Hyperinflammation similar to macrophage activation syndrome has been detected in patients with severe COVID-19, including massively increased production of cytokines, such as IL-6, IL-7, and tumor necrosis factor (TNF), and of inflammatory chemokines, such as CC-chemokine ligand 2 (CCL2), CCL3, and CXCL10 (Schulert and Grom, 2015; Mehta et al., 2020). Through scRNA-seq analysis based on bronchoalveolar fluid (BALF) from patients with COVID-19, Liao et al. found increased proportions of mononuclear macrophages accounting for 80% of total BALF cells and further identified a depletion of tissue-resident alveolar macrophages and an abundance of inflammatory monocyte-derived macrophages (Liao et al., 2020a). Moreover, a significant increase in the populations of CD14+ and CD16+ monocytes producing IL-6 was also validated in the peripheral blood samples of patients with COVID-19 through scRNA-seq analysis of peripheral blood mononuclear cells (Wen et al., 2020b). In addition, a special subset of macrophages of COVID-19 patients has been described, which is enriched in genes associated with tissue repair and promotes fibrosis generation, and scRNA-seq analysis also identified alternative M2-like macrophages that were functionally defined as a profibrotic subset (Ramachandran et al., 2019; Liao et al., 2020b). Dandan et al. (Wu and Yang, 2020) have detected high levels of IL-17-induced helper T cells (Th17 cells) in the periphery blood of patients infected with SARS-Cov-2 and those cells were considered as the main sources for IL17A which would lead to worse alveolar damage and multiple organ failures (Xu et al., 2020c). Moreover, Sodhi et al. demonstrated that ACE2 could regulate pneumonic neutrophilic infiltration through an IL17A-dependent manner in the bacterial pneumonia murine model and further found recombinant ACE2 could reduce neutrophilic infiltration by inhibiting IL17A-induced activation of STAT3 (Sodhi et al., 2019). High levels of IL18 and the reduction of mucosal-associated invariant T (MAIT) cells have also been identified in COVID-19 and significantly associated with the mortality and poor outcome in SARS-Cov-2 infection (Satis et al., 2021). Flament et al. validated the correlation between increasing cytotoxicity of circulating MAIT cells and severe inflammation response, particularly high levels of IL-18 through analysis data from 102 COVID-19 patients and 80 uninfected controls. Furthermore, these researchers proposed a two-step process of MAIT cell activation induced by type I IFN and later IL-18 through co-culture experiments of SARS-CoV-2-infected macrophages with MAIT cells *in vitro* (Gea-Mallorqui, 2020).

The T cell immune response, especially memory T cell activation, has been reported to be necessary for resolving SARS-CoV infection in patients and mice (Channappanavar et al., 2014; Liu et al., 2017). However, previous studies have certified that coronavirus could cause an irregular T cell response by stimulating T cell apoptosis (Zhou et al., 2014), and substantial clinical studies have also demonstrated a reduction in and functional exhaustion of T cells (especially CD4+ and CD8+ T cells) based on data from 522 patients with laboratory-confirmed COVID-19 (Diao et al., 2020a). In addition, during the severe and mild stages of SARS-CoV-2 infection, a lower proportion of CD8+ T cells and a higher proportion of CD4+ T cells with high levels of effector molecules were detected in severe patients than in mild patients, according to scRNA-seq analysis based on BALF from three severe and three mild COVID-19 patients (Liao et al., 2020a). Furthermore, it was also confirmed that the ratio of naïve CD4+ T cells to CD8+ T cells decreased in early recovery-stage (ERS) COVID-19 patients, while the central memory CD4+ T cells were significantly higher than in HCs by scRNA-seq analysis of peripheral blood mononuclear cells during the recovery stage of COVID-19 (Wen et al., 2020a).

Similarly, B lymphocytes, especially plasma cells, are also immune effectors that protect against viral infection through producing immunoglobulin antibodies, and for respiratory viruses, these antibodies, which reside in upper respiratory tract tissues, are particularly effective in that they provide a first line of defense against the virus at its point of entry (Sealy et al., 2013). However, the number of B cells significantly decreased in COVID-19 patients, and severe cases had a lower level than mild cases, according to clinical studies (Wang et al., 2020b). Moreover, Liu et al. also further verified that the significant decrease in B cell levels was consistent with the degree of disease severity and was detected earlier in pleural effusion than in peripheral blood *via* scRNA-seq analysis (Liu et al., 2020d). Notably, compared with that in the severe stage of COVID-19, the percentage of plasma cells increased significantly, whereas naïve B cell levels decreased significantly in the recovery stage of COVID-19 in Wen’s scRNA-seq analysis (Wen et al., 2020a).

It is well known that natural killer (NK) cells play a vital role in the control of pathogen infection by mediating cellular immunity and cytotoxic function as primary cytotoxic lymphocytes (Vivier et al., 2008). In one study, the absolute number of NK cells was found to be significantly decreased in severe and mild COVID-19 patients, and it tended to increase after remission in 32 COVID-19 patients with different severities (Jiang et al., 2020). However, Liao and her colleagues identified higher proportions of NK cells in COVID-19 patients than in controls, and severe patients contained a lower proportion of NK cells than mild patients, according to scRNA-seq analysis (Liao et al., 2020a). Regarding the recovery stage of COVID-19, the proportion of NK cells was found to be higher than that in the HCs, and these cells express higher levels of inflammatory genes. In contrast, during the later recovery stage, these patients have an increase in NK cell levels with lower expression of inflammatory genes (Wen et al., 2020b).

During the progression of SARS-CoV-2 infection, innate and adaptive immune responses are activated, with massive inflammatory factor release, which causes exhaustion of various types of immune cells, especially lymphocytes (Xiong et al., 2020). The recent research provides data of adaptive immunity in COVID-19 patients (Schultheiss et al., 2020). The application of NGS technology, including scRNA-seq analysis, clearly revealed the landscape of immune cells during the different stages of COVID-19, which could be a valuable resource for clinical guidance on anti-inflammatory medication and understanding the molecular mechanisms.

## Conclusion

COVID-19 has pushed the world to the brink. Its outbreak has seriously threatened public health and caused general panic. This is perhaps one among the first reviews that focuses on the application of NGS to COVID-19. NGS, the ultra-rapid and cost-effective technology, has contributed to progress in determining novel SARS-CoV-2 hosts, evolution, and spread patterns, which are critical to designing effective disease control and prevention strategies. The rate of data acquisition and analysis has been inconceivably rapid which made the NGS methods applicable to monitor and counter the spread of SARS-CoV-2. Coordinated efforts are required to promote the principles of data sharing in order to facilitate more efficient data analysis of SARS-CoV-2.

In just very short time, NGS was applied to reveal the expression features of ACE2 in different organ systems and specific cells after infection with SARS-CoV-2 and to characterize the intestinal flora. This map might provide potential clues for interpreting different organ damage due to SARS-CoV-2 infection and help doctors better prevent target organ damage in the clinic, indicating the essential role of sequencing technology in filling the gap between molecular research and clinical application. Moreover, these findings are expected to provide further insight into the role of ACE2 and the pathogenesis of SARS-CoV-2 infection.

The importance of the intestinal flora in COVID-19 infection has been recognized and accepted by the Chinese government and the majority of frontline medical staff. Although there are currently no specific antiviral treatments, we speculate that probiotics can regulate the intestinal flora, thereby beneficially changing gastrointestinal symptoms and providing respiratory protection. Further study should focus on investigating whether the benefits of ACE2 for lung diseases may be mediated by regulating the intestinal and/or lung microbiota. The profiled immune cells also provide insight into adaptive immunity to SARS-CoV-2 for therapeutic guidance and vaccine development.

In conclusion, the application of NGS is crucial to understand the pathophysiologic mechanisms underlying the systemic manifestations of COVID-19 and its clinical significance. The insights from this review will add new dimensions to the understanding of infectious diseases and will benefit future decisive actions.

## Author Contributions

SJ, XL, and JW conceived this review. XC organized and critically revised the manuscript. YK and JL did major work of writing the manuscript. KP and XX made contributions to writing the manuscript. All authors contributed to the article and approved the submitted version.

## Funding

This work was supported by The Key Scientific and Technological Innovation Projects of Wenzhou (grant ZY202002) and Chinese Academy of Engineering Fund (2020-KYGG-02-02).

## Conflict of Interest

The authors declare that the research was conducted in the absence of any commercial or financial relationships that could be construed as a potential conflict of interest.

## Supplementary Material

Supplementary Figure 1
**(A)** The cumulative number of confirmed cases, as well as the cumulative number of cured and deaths around the world. **(B)** Number of patients in the top 25 countries and China with the largest number of confirmed cases. **(C)** Cure and deaths rates in the top 25 countries and China with the largest number of confirmed cases. The data comes from the website which is daily updated. (https://voice.baidu.com/act/newpneumonia/newpneumonia/?from=osari_aladin_banner#tab4)
