# Supplementary figures and images for: Next-Generation Sequencing Reveals the Progression of COVID-19
Source: Front Cell Infect Microbiol. 2021 Mar 11;11:632490. doi: 10.3389/fcimb.2021.632490 (PMC7991797; doi:10.3389/fcimb.2021.632490)

**A**

Cumulative diagnosis : 78939299

Cumulative cure: 55246193

Cumulative deaths: 1731883

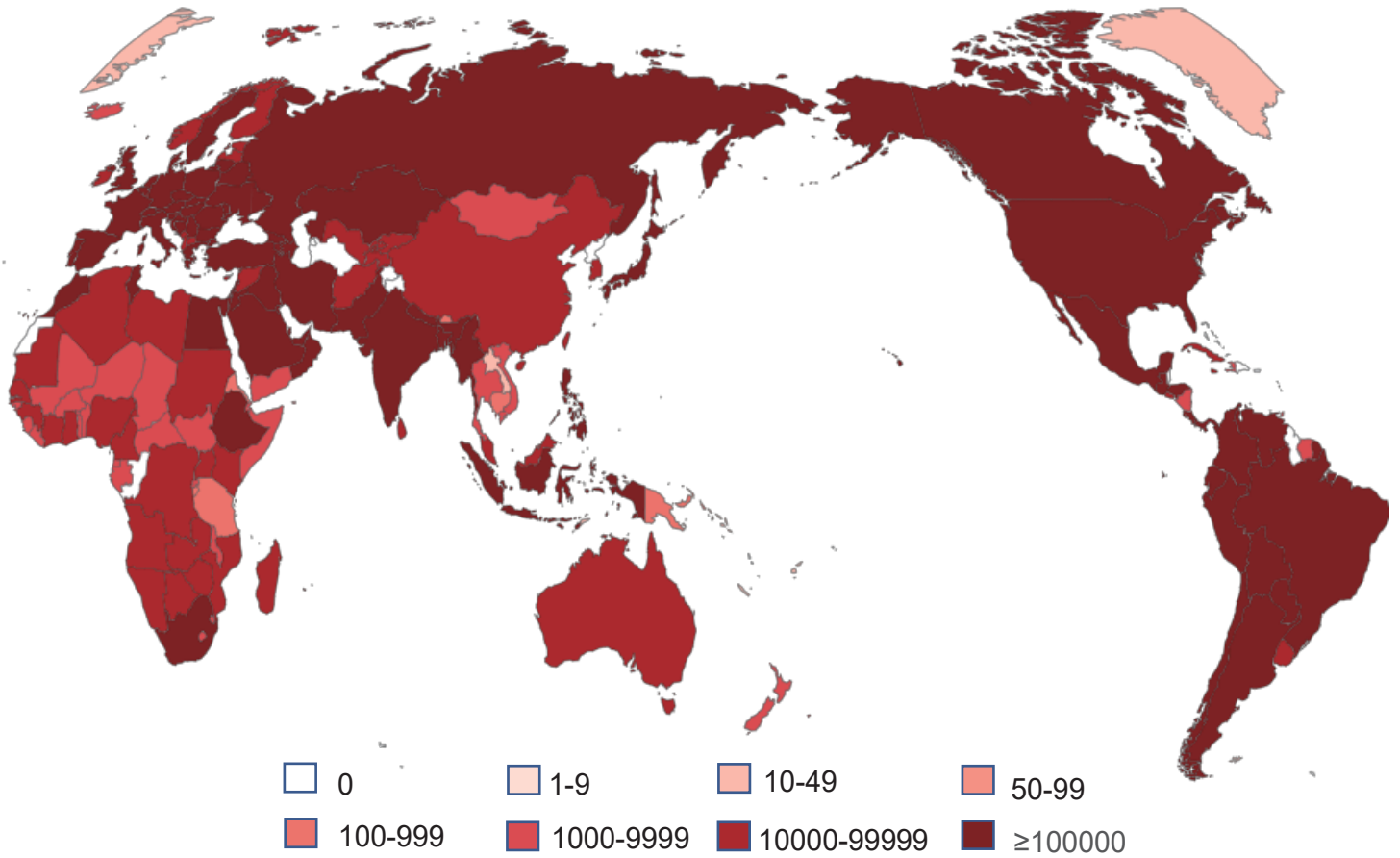

**B**

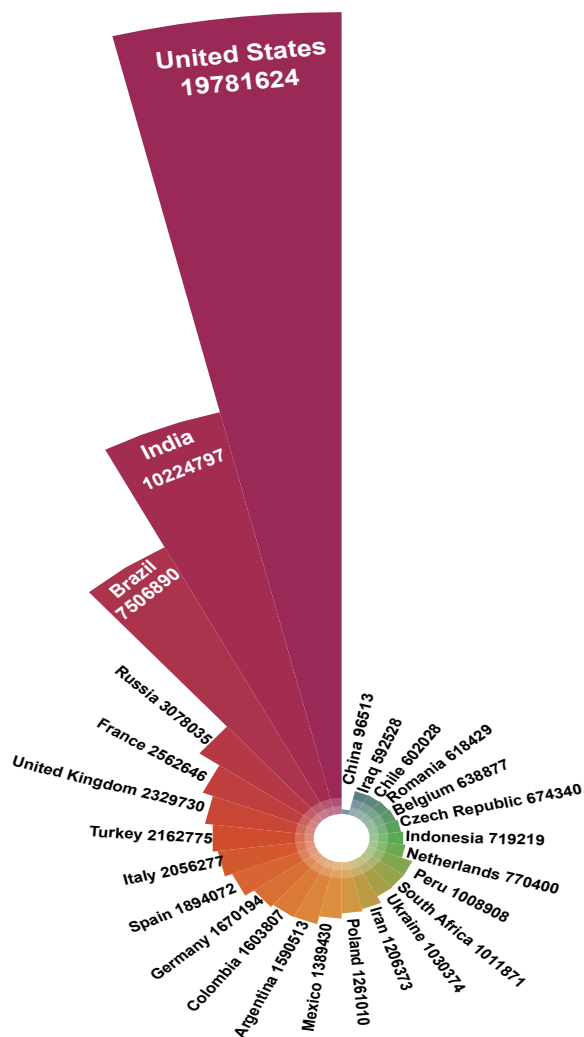

**C**

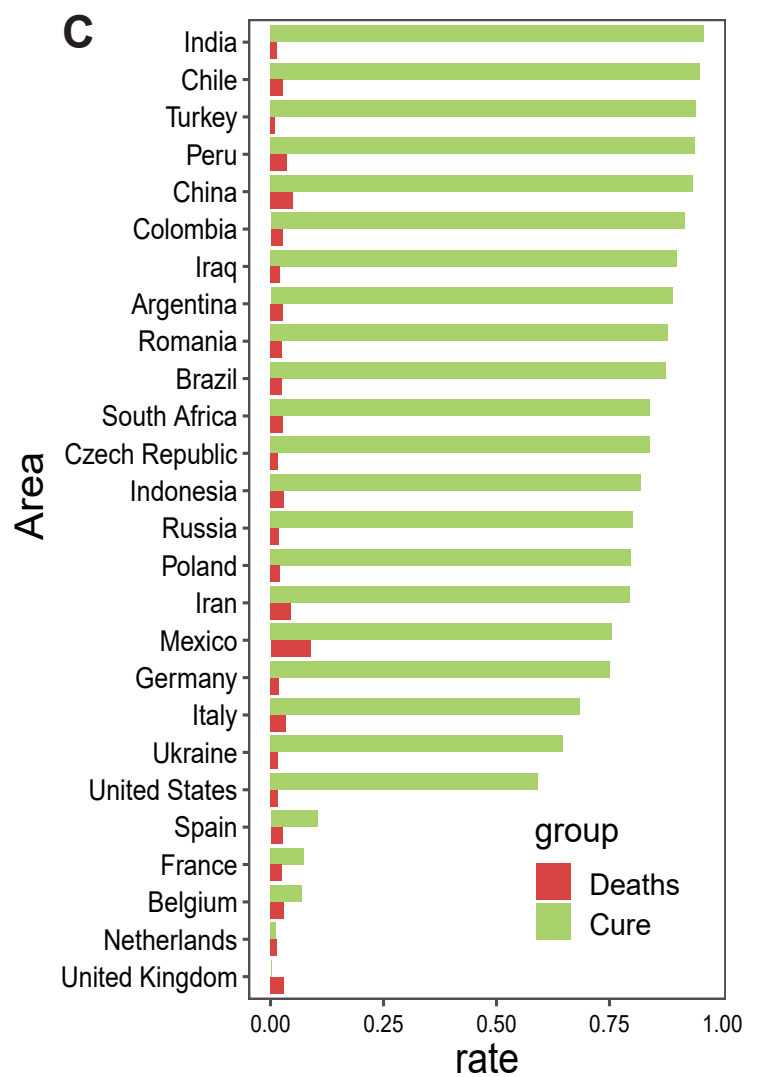

Supplement: Supplementary Figure 1 — (A) The cumulative number of confirmed cases, as well as the cumulative number of cured and deaths around the world. (B) Number of patients in the top 25 countries and China with the largest number of confirmed cases. (C) Cure and deaths rates in the top 25 countries and China with the largest number of confirmed cases. The data comes from the website which is daily updated. (https://voice.baidu.com/act/newpneumonia/newpneumonia/?from=osari_aladin_banner#tab4) [file Image_1.pdf]
