# Supplementary material for: Next-Generation Sequencing Reveals the Progression of COVID-19
Source: Front Cell Infect Microbiol. 2021 Mar 11;11:632490. doi: 10.3389/fcimb.2021.632490 (PMC7991797; doi:10.3389/fcimb.2021.632490)
Supplement: Supplementary file 2 [file DataSheet_1.pdf]

Supplementary Table 1 Blast result in partial protein sequence of PsNV genome and SARS-CoV-2 genome

genome1_region	genome2_region	% identity
PsNV_1(2959-3314)	SARS-CoV-2_1(3241-3575)	25.5
PsNV_2(3822-4326)	SARS-CoV-2_2(3892-4403)	22.6
PsNV_3(82-2266)	SARS-CoV-2_3(4488-6801)	34.8
PsNV_4(2454-2716)	SARS-CoV-2_4(6824-7082)	31.6
